# Authors’ Responses to Peer Reviews of “Exercise-Induced Hypoalgesia Following Proprioceptive Neuromuscular Facilitation and Resistance Training Among Individuals With Shoulder Myofascial Pain: Randomized Controlled Trial”

**DOI:** 10.2196/45038

**Published:** 2022-12-27

**Authors:** Zi-Han Xu, Nan An, Zi-Ru Wang

**Affiliations:** 1 School of Sport Medicine and Rehabilitation Beijing Sport University Beijing China

**Keywords:** exercise induced hypoalgesia, proprioceptive neuromuscular facilitation, PNF, resistance exercise, conditioned pain modulation, myofascial pain syndrome, resistance training, hypoalgesia, exercise-induced hypoalgesia, shoulder myofascial pain, myofascial pain, pain management, chronic pain, musculoskeletal pain, physical therapy, physiotherapy, shoulder pain, upper back pain, exercise, pain

*This is the authors’ response to peer-review reports for “Exercise-Induced Hypoalgesia Following Proprioceptive Neuromuscular Facilitation and Resistance Training Among Individuals With Shoulder Myofascial Pain: Randomized Controlled Trial”*

## Round 1 Review

### Responses to Comments From Reviewer AC [1]

#### General Comments

This paper [2] set to estimate the effect of proprioceptive neuromuscular facilitation (PNF) and resistance training on exercise-induced analgesia and conditioned pain modulation among young adult women with myofascial pain syndrome.

The paper holds several strengths, including random assignment and the inclusion of PNF, 2 resistance training exercise types, and 1 passive control group, which enables comparison across exercise conditions. Authors justifiably correct for multiple comparisons. The discussion thoroughly interprets the findings and relates them to existing literature. Some questions and potential limitations are listed below.

Response:

We appreciate this supportive feedback.

#### Major Comments

1. The study is limited to young women (18-30 years old) and therefore has limited generalizability to men, as well as women above the age of 30 years. Authors partially acknowledge this in the limitations section (with regard to gender).

Response:

Thank you for raising this point. We adjusted statements about gender and age in the limitations section. In our university, female students were the most common group of shoulder and neck pain considering the gender difference in pain perception and sedentary behaviors, so we enrolled female students to enhance the comparability of the results.

2. The sample size in each group is modest (n=18-20), limiting statistical power.

Response:

Thanks for your comment. We agree that the sample size is relatively small and may attenuate the statistical power. Since this is a pilot study aiming at investigating the potential relationship, and given the feasibility issues during the national COVID-19 prevention and control policies in our setting, we were trying to recruit as many participants as we could to meet the basic requirement to appeal the effect size. We added more description about the sample size calculation in the participant section.

3. Did the authors have a specific hypothesis about the relative effect of PNF, isometric, and isotonic exercise training on outcomes? Such a hypothesis is now stated. Was the testing of differential effects exploratory?

Response:

Thank you for your valuable and considerate suggestion. Considering the recent progress [3] in the central endogenous pain modulation, we found that the different C afferent fibers’ input can trigger the descending inhibition or facilitation pathway. In the context of resistance exercise, the concentric or eccentric muscle contraction with a subpain threshold may activate the nonnoxious C fibers and induce pain inhibition. Thus, we developed and added our preliminary hypothesis in the discussion section and revised the mechanisms of endogenous pain modulation in the introduction section.

4. Authors indicate that “Randomized sequences were generated by computer.” Can authors provide details on the method, software, or website used for randomization?

Response:

Thank you for the above suggestions. All participants were labeled from number 01 to 76, then the sequence was randomized using Excel (Microsoft Corp), and allocated following the A-B-C-D circulation order. We added the detailed method of randomization in the participant section.

5. Authors indicate that participants were excluded if they experienced depression, psychosis, cognitive impairment, etc. How were these assessed?

Response:

Thank you for raising this important point. During the screening of participants, our lab employed a physician who was responsible for evaluating the medical risks for all participants, including depression, psychosis, and cognitive impairment. The self-rating depression scale, self-rating anxiety scale, and the brief psychiatric rating scale were also applied and assessed by the physician during the screening periods.

#### Minor Comments

6. Authors make use of 6 different acronyms in the abstract, which may make it more difficult to read, particularly for individuals outside this immediate field. When possible, consider spelling things out to increase ease of readability.

Response:

We apologize for the lack of clarity. We reduced the acronyms as far as we can in the revised manuscript. Only 3 acronyms including “PNF,” “CPM,” and “EIH” were retained.

7. Please change all instances of “*P*<.000” to “*P*<.001”

Response:

Thank you so much for your careful check; we have changed all reports about the *P* values according to your and our editors’ comments.

8. There are some typos throughout the manuscript, please correct those (eg, “Our findings mostly met what we previously hypothesized, where was an increase in PPT at trigger point”; which may have been “There was an increase,” or “Crombie et al investigated that the serum endocannabinoids increased,” which may have been “Crombie et al ‘found’ or ‘reported’ that….,” as well as other examples throughout).

Response:

We apologize for the typos. We double-checked and revised all typos and grammar errors in the manuscript.

### Responses to Comments From Reviewer Anonymous [4]

#### General Comments

This paper [2] aims to compare short-term exercise-induced hypoalgesia responses following different types of exercise in pain modulation within patients with myofascial pain. It is generally well written and presents innovative results to clarify the knowledge in the treatment of myofascial pain.

Response:

We thank the reviewer for reading our paper carefully and for the encouraging feedback.

#### Major Comments

1. Methods: In the procedures section, please add information about possible blinding of the evaluators (ie, experience of the persons who did the manual assessment of the myofascial pain syndrome, people who performed the exercise programs, etc).

Response:

Thank you for raising this point. The allocation of researchers was strictly carried out following the double-blinded principle, and we separated evaluation and intervention across different researchers. We have added all information about researchers in the procedure section.

#### Minor Comments

2. Discussion: Please try to address the important improvements in the proprioceptive neuromuscular facilitation exercise group in relation to personal interaction with the researcher (manual contact, personal adaptation, etc).

Response:

Thanks for your valuable comment; we agree that the interaction between the participants and the physical therapist has positive effects on proprioception processing and pain management. We have addressed this mechanism at the end of the “potential mechanism of EIH” part in the Discussion section.

### Responses to Comments From Reviewer DA [5]:

#### General Comments

This paper [2] shows the effects of several types of exercise on exercise-induced hypoalgesia and conditioned pain modulation in patients with myofascial pain syndrome (MPS) of upper trapezius muscle. I thoroughly reviewed this work and find that there is room for improvement. The comments are as follows.

Response:

We thank the reviewer for reading our paper carefully and giving the above positive comments.

#### Major Comments

1. Why was this sample size chosen?

Response:

Thank you for pointing this out. According to the pressure pain threshold (PPT) changes seen in previous studies, we used G-Power software with *F* test and ANCOVA parameters to calculate the sample size. The total samples of this study should be a minimum of 76 participants in the 3 groups, or 19 participants in each group. We added these sample size calculation methods into the participant section.

2. Regarding the first inclusion criteria, the patients must report their MPS for at least 4 weeks up to 3 months. In my opinion, the patients do not know whether they have MPS, and they commonly complain about their shoulder pain only. They will be informed about having MPS after being diagnosed by the physicians. Thus, it is not clear what the statement, “reported MPS” is referring to. In addition, I am not sure if patients with cardiovascular conditions, such as uncontrolled hypertension, are excluded from the study. Is it because of some risks of cardiovascular problems while performing accidental events during exercises such as holding the breath or due to resistant exercise–induced cardiovascular problems?

Response:

We gratefully thank you for the precious time your spent making constructive remarks.

First, we are very sorry for the inaccurate writing of the inclusion criteria, and it should be “reported shoulder pain” in practice. During the recruitment period, we brought in all participants reporting shoulder pain, then carefully evaluated their symptoms following the criteria of the upper trapezius MPS. Second, our lab employed a physician who was responsible for evaluating the medical risks for all participants, including cardiovascular problems during the screening periods. Participants who presented hypertension or other risky cardiovascular conditions have been excluded from the study.

3. I am not sure how 60% maximum voluntary contraction (MVC) or pain-free load is set for each exercise intervention. Can we set it with other intensities? Is it possible that the patients doing an exercise with 60% MVC or pain-free load show significant differences on the outcomes? Why does the proprioceptive neuromuscular facilitation (PNF) method use 60% MVC for designing the PNF training protocol? The PNF intensity is set as a maximal resistance of the patients to facilitate an optimal outcome. Some techniques, such as the hold-relax technique with maximal resistance but within subpain threshold, are effectively used for relaxing muscle spasm or guarding in patients with muscle pain conditions. The authors apply agonist reversal, combination of isotonic contraction, and rhythmic stabilization without providing details of start and end positions, thus making it difficult to follow.

Response:

Thank you for your valuable and constructive comments. (1) Considering the previous EIH studies, the optimal intensity of resistance exercise in patients with chronic pain may not be 100% MVC or over-pain thresholds, and it may elicit pain symptoms during the exercise [6]. In most cases, we may prefer exercise with subpain threshold intensity (around 50%-60% MVC) in the context of pain management [7]. (2) For the PNF intervention, it is true that the maximal resistance can facilitate an outcome about flexibility and the joint range of motion. However, some previous studies [8,9] compared the analgesic effects of PNF hold-relax methods with different intensities and found that exercise with 60%-70% MVC may be more suitable for pain treatment. In our study, the intensity of exercise was set as 60% MVC, and then it could adjust to subpain thresholds if participants reported pain during the exercise. (3) In addition, as the reviewer suggested, we have rewritten the PNF method section and added practical details in the agonist reversal, combination of isotonic contraction, and rhythmic stabilization training, including the joint positions and movements.

#### Minor Comments

1. Please provide a specific name for a muscle affected by MPS, such as “patients with MPS of upper trapezius muscle.”

Response:

Thank you for pointing this out. We specified the upper trapezius as the MPS-affected muscles in the Abstract and Methods sections.

2. Exercise-induced hypoalgesia should be defined in relation to what outcomes are included for it.

Response:

Thank you for the above suggestions. We defined the improvement of PPTs as the EIH response.

3. Please specify specific area of pressure pain threshold of remote sites on the extensor carpus radialis and the peroneus longus.

Response:

Thank you for raising this concern. We have added the specific test point of the extensor carpus radialis (5 cm below the lateral condyle of humerus) and the peroneus longus (10 cm below the lateral femoral condyle) in the outcome measure section.

4. Is the visual analog scale one of the study's outcomes?

Response:

No. The visual analog scale would be only measured as the baseline characteristics and would not be considered as an outcome measurement. We moved the visual analog scale–related parts to the Procedures section.

## Abbreviations

EIH: exercise-induced hypoalgesia
MPS: myofascial pain syndrome
MVC: maximum voluntary contraction
PNF: proprioceptive neuromuscular facilitation
PPT: pressure pain threshold

## References

[1] Greenberg J (2022). Peer Review of “Exercise-Induced Hypoalgesia Following Proprioceptive Neuromuscular Facilitation and Resistance Training Among Individuals With Shoulder Myofascial Pain: Randomized Controlled Trial”. JMIRx Med.

[2] Xu ZH, An N, Wang ZR (2022). Exercise-Induced Hypoalgesia Following Proprioceptive Neuromuscular Facilitation and Resistance Training Among Individuals With Shoulder Myofascial Pain: Randomized Controlled Trial. JMIRx Med.

[3] You HJ, Lei J, Pertovaara A (2022). Thalamus: The 'promoter' of endogenous modulation of pain and potential therapeutic target in pathological pain. Neurosci Biobehav Rev.

[4] Anonymous (2022). Peer Review of “Exercise-Induced Hypoalgesia Following Proprioceptive Neuromuscular Facilitation and Resistance Training Among Individuals With Shoulder Myofascial Pain: Randomized Controlled Trial”. JMIRx Med.

[5] Areeudomwong P (2022). Peer Review of “Exercise-Induced Hypoalgesia Following Proprioceptive Neuromuscular Facilitation and Resistance Training Among Individuals With Shoulder Myofascial Pain: Randomized Controlled Trial”. JMIRx Med.

[6] Coombes BK, Wiebusch M, Heales L, Stephenson A, Vicenzino B (2016). Isometric Exercise Above but not Below an Individual's Pain Threshold Influences Pain Perception in People With Lateral Epicondylalgia. Clin J Pain.

[7] Vaegter HB, Handberg G, Graven-Nielsen T (2014). Similarities between exercise-induced hypoalgesia and conditioned pain modulation in humans. Pain.

[8] Lim W (2018). Optimal intensity of PNF stretching: maintaining the efficacy of stretching while ensuring its safety. J Phys Ther Sci.

[9] Kwak DH, Ryu YU (2015). Applying proprioceptive neuromuscular facilitation stretching: optimal contraction intensity to attain the maximum increase in range of motion in young males. J Phys Ther Sci.

